# Association Between Diet and Emotional Symptoms in Early Childhood: Cross-Sectional Results from the Piccolipiù Cohort

**DOI:** 10.3390/nu17182909

**Published:** 2025-09-09

**Authors:** Federica Concina, Giulia Zamagni, Eleonora Maurel, Claudia Carletti, Alessandra Knowles, Martina Culasso, Franca Rusconi, Maja Popovic, Luca Ronfani, Lorenzo Monasta, Deborah N. Ashtree, Paola Pani

**Affiliations:** 1 Clinical Epidemiology and Public Health Research Unit, Institute for Maternal and Child Health-IRCCS Burlo Garofolo, 34137 Trieste, Italy; federica.concina@burlo.trieste.it (F.C.); claudiaveronica.carletti@burlo.trieste.it (C.C.); alessandra.knowles@burlo.trieste.it (A.K.); luca.ronfani@burlo.trieste.it (L.R.); lorenzo.monasta@burlo.trieste.it (L.M.); paola.pani@burlo.trieste.it (P.P.); 2 Department of Epidemiology, Lazio Regional Health Service-ASL ROMA1, 00154 Rome, Italy; m.culasso@deplazio.it; 3 Department of Mother and Child Health, Azienda USL Toscana Nord Ovest, 56121 Pisa, Italy; franca.rusconi@uslnordovest.toscana.it; 4 Cancer Epidemiology Unit, Department of Medical Sciences, University of Turin, 10126 Turin, Italy; maja.popovic@unito.it; 5 IMPACT (The Institute for Mental and Physical Health and Clinical Translation), Food & Mood Centre, School of Medicine, Barwon Health, Deakin University, Geelong 3220, Australia; debbie.ashtree@deakin.edu.au

**Keywords:** children, dietary habits, anxiety, depression, CBCL, food groups

## Abstract

Background/Objectives: Emerging evidence suggests the critical role of diet in shaping mental health outcomes, which are increasingly prevalent among children and adolescents worldwide. This study aims to investigate whether the dietary habits of children in the Italian multicenter Piccolipiù birth cohort at four years of age were associated with anxiety and depression symptoms. This analysis was conducted within the framework of the Global burden of disease Lifestyle And mental Disorder (GLAD) Project (DERR2-10.2196/65576). Methods: Data from 1726 children were analyzed. Emotional symptoms were assessed with the Child Behavior Checklist (CBCL), and diet was assessed using a food frequency questionnaire. Sociodemographic data, including per capita income, were also collected. Associations between food intake (grams per day) and clinical anxiety/depression (T-scores > 70) or total symptom scores were examined using univariate and multivariable logistic and robust linear regressions adjusted for sex and income. Results: In 1726 children (median age 4.4 years; 50% female), 3% exhibited clinical anxiety and 2% clinical depression. Higher grain intake was associated with increased odds of clinical anxiety (OR = 1.004; 95% CI: 1.001–1.007), while greater fish consumption reduced odds of clinical depression (OR = 0.946; 95% CI: 0.903–0.992). Vegetable intake was associated with lower anxiety and depression scores. Multivariable analyses confirmed that grain intake is positively associated with anxiety, while fish consumption is inversely associated with depression. Conclusions: Higher intake of vegetables, fruits, and fish may be associated with better emotional health in preschoolers, although effect sizes were modest. Early dietary interventions may offer a practical approach to improving children’s long-term mental health. Longitudinal studies are needed to confirm these associations and clarify underlying mechanisms.

## 1. Introduction

Emerging evidence suggests the critical role of diet in shaping mental health outcomes, particularly anxiety and depression [1,2], which are increasingly prevalent among children and adolescents worldwide [3]. Depression and anxiety arise from a combination of various complex interconnected environmental and genetic factors, and their precise etiology remains unclear. Studying the role of modifiable risk factors such as diet is important for intervention [2,4].

Childhood and adolescence are pivotal periods for brain development and emotional regulation, during which nutritional intake can exert profound influences on cognitive functions, learning capacity, and emotional stability. Much of the existing literature has focused on the detrimental effects of ultra-processed foods on pediatric mental health, but little evidence is available on the impact of specific food groups on anxiety and depression [2]. Findings from observational studies and meta-analyses suggest that healthy dietary patterns, characterized by high consumption of fruit, vegetables, whole grains and fiber and low intake of saturated fats, during the prenatal, childhood, and adolescent stages, are associated with reduced risk of internalizing (e.g., anxiety, depression) and externalizing (e.g., attention-deficit/hyperactivity disorder, conduct disorder) disorders [4]. In contrast, diets rich in sugars, saturated animal fats and ultra-processed foods, combined with low intakes of nutrient-dense foods, are linked to increased risk of anxiety and depressive symptoms [4].

Moreover, quantitative dietary indices, such as the Mediterranean Diet Score, have revealed significant associations between low adherence to Mediterranean diet and both clinical diagnoses and symptom severity of depression and anxiety in adult populations (18–65 years). Specifically, analyses focusing on individual food groups indicate that higher intake of vegetables is inversely correlated with symptom severity for depression, anxiety, and fear [5]. Similarly, increased consumption of non-refined grains is associated with reduced severity of depression and anxiety symptoms and lower odds of clinical diagnosis, even after controlling for other dietary factors [5].

The association between diet and depressive symptoms in early childhood has not yet been adequately addressed in the literature. This relationship is complex and influenced by multiple behavioral, psychosocial, and socioeconomic factors that include maternal diet and parental mental health, child physical activity levels, screen time, and broader social determinants. The quality of maternal diet during pregnancy has been associated with a range of developmental outcomes across cognitive, behavioral and emotional domains in the offspring [6,7,8]. Maternal depressive symptoms have been associated with less healthy parenting behaviors, which can negatively impact children’s dietary habits and mental health outcomes [9,10]. Parental anxiety and depression have been linked to increased food fussiness in children, which may act as an intermediary risk factor for internalizing disorders [11]. Regarding physical activity and depressive symptoms in children, there is evidence that children with internalizing disorders tend to engage in significantly lower levels of physical activity [12]. Moreover, excessive screen time has been linked to higher risks of anxiety and depression in children, particularly during periods of restricted movement, such as during the COVID-19 pandemic [13,14]. Socioeconomic status remains a significant determinant of both diet quality and mental health. Lower socioeconomic status is frequently associated with suboptimal dietary patterns and higher prevalence of depressive symptoms among children and adolescents [15].

This study aims to investigate whether the dietary habits of children in the Italian Piccolipiù birth cohort at four years of age are associated with early emotional outcomes, specifically focusing on symptoms of anxiety and depression. This analysis was conducted within the framework of the Global burden of disease Lifestyle And mental Disorder (GLAD) Project (DERR2-10.2196/65576), whose objective is to gather evidence on the association between lifestyles and Common Mental Disorders (CMDs) to support the informed inclusion of risk factors attributable to CMDs in the Global Burden of Disease (GBD) Study framework [16].

## 2. Materials and Methods

This study utilizes data from the Italian multicenter Piccolipiù birth cohort, designed to evaluate the effects of prenatal and early postnatal environmental, parental, and social exposures on child health and development. Piccolipiù recruited 3358 mother–child pairs from six maternity units across five Italian cities (i.e., Florence, Rome, Trieste, Turin, and Viareggio) between October 2011 and March 2015. All singleton pregnant women giving birth in one of the selected maternity units were considered eligible for recruitment if they met the following criteria: being at least 18 years old; residing within the catchment area of the participating maternity centers; having sufficient knowledge of the Italian language to understand the informed consent process and complete the study questionnaires; and able to be contacted via at least one telephone number. Follow-up assessments were conducted at 6, 12, 24, 48, and 84 months postpartum. Further details on the Piccolipiù study protocol are available elsewhere [17].

The 4-year follow-up assessment was carried out in the framework of the Piccolipiù in Forma project and had a special focus on obesity, nutrition, and physical activity (https://piccolipiuinforma.it/ accessed on 1 September 2025). During this follow-up visit, children were also assessed for their psychomotor and cognitive development and for emotional and behavioral problems appraised using a validated caregiver report, the Child Behavior Checklist (CBCL) 1.5–5 years [18]. Overall, the response rate was 64.9% for the dietary questionnaire and 61.4% for the CBCL.

### 2.1. Exposure

Dietary intake evaluated at the 4-year follow-up used a food frequency questionnaire (FFQ), compiled by the parents based on the child’s habitual diet during the previous two months of follow-up visit. The FFQ collected information on frequency of consumption and portion size of 46 different food items using the Scotti-Bassani pediatric food portions atlas [19]. Dietary exposure was defined in accordance with GBD. For each child, the frequency of consumption in grams per day of each item was calculated, combining food portions and frequencies as follows: Portion in grams per day = Portion size (g) × times per month/30.4 OR portion size (g) × times per week/7 OR daily size (g) × times/day. The average daily intake expressed in grams per day was calculated for each of the following food groups: vegetables, fruit, legumes, grains, milk, red meat, processed meat, fish, and sugar-sweetened beverages.

### 2.2. Outcome

The CBCL provides total scores and standardized scores (i.e., T-scores, with mean = 50 and standard deviation = 10) for a wide range of behavioral and emotional problems in children. Total scores are calculated as the sum of item scores for each sub-scale. Each item is rated by a parent or caregiver as follows: 0 = not true; 1 = somewhat or sometimes true; 2 = very true or often true. T-scores are obtained by converting raw scores using normative tables based on large-scale population samples of children matched by age and sex [20]. The present study was specifically focused on anxiety and depression, based on (i) clinical classification, where children were classified as having clinically significant anxiety or depression if their standardized T-scores exceeded 70, following established CBCL guidelines [20], and (ii) raw total scores, expressed as continuous measures, to capture the full spectrum of symptom severity, including subclinical levels. As the anxiety and depression subscales include ten items each, total scores can range from 0 to 20.

### 2.3. Covariates

Moreover, sociodemographic data were collected, and family income was used as a proxy for socioeconomic status. Monthly family income was originally recorded using predefined categories: less than EUR 1000; EUR 1000–1499; EUR 1500–1999; EUR 2000–2499; EUR 2500–2999; EUR 3000–3999; EUR 4000–4999; EUR 5000–5999; and EUR 6000 or more. An additional category indicating an inability to quantify the income was also included. The categorical income variable was transformed into a continuous one by assigning the median value of each bracket [16]. For the open-ended lowest and highest categories, the respective threshold value was assigned. Cases with unquantifiable income were excluded from analyses. To obtain a more individualized measure of economic capacity and allow fairer comparisons across households of different sizes, monthly family income was divided by the number of household components to compute per capita income, expressed in Euros.

Therefore, the present analysis included all children with available data for the FFQ, the CBCL, and family income at the 4-year follow-up.

### 2.4. Statistical Analyses

Categorical variables were summarized as absolute frequencies and percentages, while continuous variables were presented as median and interquartile range (IQR), given the non-normal distribution confirmed by the previously performed Shapiro–Wilk normality test.

Food group consumption was compared between children with and without clinical anxiety, and between those with and without clinical depression, using the Wilcoxon–Mann–Whitney test.

Univariate logistic analyses were conducted to identify potential dietary risk factors for both clinical anxiety and clinical depression, with odds ratios (ORs) reported alongside 95% confidence intervals (CIs).

Furthermore, multivariable analyses were carried out to explore the associations between food group consumption and mental health outcomes, adjusting for potential confounders (i.e., sex and per capita income). Specifically, logistic regression models were applied to binary clinical outcomes, while robust linear regression models based on MM-estimators were used for raw scores. This approach was adopted to address the skewness of the dependent variables, which can result in non-normal residuals and violate key assumptions of ordinary least squares regression. By relaxing the requirement for normally distributed residuals and reducing sensitivity to outliers and heteroscedasticity, robust regression offers more stable and reliable estimates under these conditions. All estimates (i.e., OR and regression coefficients) were reported with 95% CI. ORs and regression coefficients were presented per gram-per-day increase in the consumption of each food group. The Benjamini–Hochberg procedure was applied to control the false discovery rate (FDR), considering the large number of multiple comparisons conducted. All statistical analyses were performed using R4.1.2., with statistical significance set at *p* < 0.05 (R Core Team (2021). [21] R: A language and environment for statistical computing. R Foundation for Statistical Computing, Vienna, Austria. URL https://www.R-project.org/).

## 3. Results

A total of 2166 dietary questionnaires and 2062 CBCL questionnaires were completed, with 2004 children providing data for both. Of these, 1886 were retained for analysis as they were 4 years old at the time of assessment, while children who completed the follow-up at age 5 were excluded. Among the 1886 participants, 1855 had available data on family income, although only 1726 provided quantifiable information (excluding those who reported being unable to estimate their income) (Figure S1). Therefore, the final sample included 1726 children (median age: 4.4 years, IQR: 4.2–4.5), 50% of whom were female. The median per capita monthly income was EUR 687.4 (IQR: €562.4–916.5) (Table 1).

**Figure 1:**
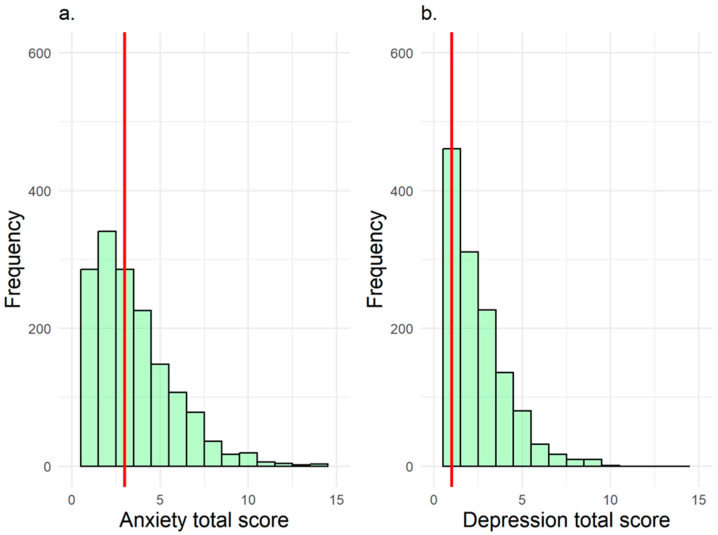
Distribution of total raw scores for anxiety (**a**) and depression (**b**). Red vertical lines represent median total scores.

Overall, 3% of children exhibited clinically significant anxiety symptoms, and 2% showed clinically significant depressive symptoms. Median CBCL raw scores were 3 (IQR: 1–4) for anxiety and 1 (IQR: 0–3) for depression (Figure 1).

Daily intake of each food group, stratified by clinical anxiety and depression status, is reported in Table S1. In univariable analysis, grain intake was associated with increased odds of clinical anxiety (OR = 1.004; 95% CI: 1.001–1.007), while for clinical depression, higher fish consumption was associated with reduced odds of symptoms (OR = 0.946; 95% CI: 0.903–0.992), and no associations were observed for other food groups.

When analyzing total scores, only vegetable intake was associated with lower anxiety (β = −0.004; 95% CI: −0.007 to −0.001) and depression scores (β = −0.003; 95% CI: −0.006 to −0.001) (Table S2). No other food groups were associated with anxiety or depression total scores.

Multivariable analyses for anxiety, adjusted for sex and per capita income, are presented in Table 2. Greater grain consumption was associated with anxiety, with more evident association when considered as clinical anxiety (OR = 1.004; 95% CI: 1.001–1.007). However, after controlling for FDR, there was no evidence of significant associations between anxiety and any of the food groups.

Regarding depression total scores, higher intake of both fruit and vegetables corresponded to lower depressive symptomatology (β = −0.002; 95% CI: −0.003 to −0.0001; β = −0.003; 95% CI: −0.005 to −0.001) (Table 3a). No other food groups showed associations with depression total scores. For clinical depression, fish consumption was associated with reduced odds of symptoms (OR = 0.951; 95% CI: 0.907–0.997). No other dietary factors were associated with clinical depression (Table 3b). After controlling for the FDR, only vegetable consumption remained significantly associated with total depression scores, whereas no significant associations were confirmed between clinical depression and any of the food groups.

## 4. Discussion

This study examined the associations between food groups and internalizing mental health outcomes in a large multicenter cohort of Italian preschool-aged children, using both clinical cutoffs and continuous measures from the CBCL. Overall, a nuanced association was found between certain dietary intakes and symptoms of anxiety and depression. As expected, the prevalence of clinically meaningful symptoms was relatively low, with 3% of children meeting criteria for anxiety and 2% for depression. These rates are consistent with international epidemiological data that suggest that clinical manifestations of internalizing disorders are less common in early childhood compared to later developmental stages [2]. The multivariable analyses, adjusted for sex and per capita income, revealed several noteworthy findings. Grain consumption was consistently and positively associated with clinical anxiety, but no association was found with raw anxiety scores. This pattern may reflect a threshold effect, whereby higher levels of grain intake are associated with clinically significant symptoms but not with subclinical variations, or may be attributable to the low outcome prevalence (3%). The consumption of refined grains, which are often characterized by high glycemic load and limited nutritional value compared with whole grains, has been linked to systemic inflammation, oxidative stress, and increased risk of depression and anxiety in the adult population [5,22]. In the current study we only had data on total grain consumption. It would have been interesting to have data distinguishing between whole and refined grain intake to clarify their respective impacts on child mental health. Moreover, after adjustment for false discovery rate, the association between grain consumption and anxiety was no longer statistically significant, most likely reflecting limited statistical power given the low prevalence of the outcome. Regarding depression, the multivariable analyses revealed that fruit and vegetable consumption was associated only with depressive symptoms as reflected by total scores, suggesting that regular intake of these foods may offer subtle but consistent emotional benefits. Since fruits and vegetables are rich in fibers, vitamins (e.g., folate, vitamin C), antioxidants, and anti-inflammatory compounds, they can reduce oxidative stress factors, which are increasingly recognized in the pathophysiology of depression, and may contribute to improved mood regulation, as observed in the adult population. Importantly, the association we observed concerned only subclinical depressive symptoms, and not clinical depression, possibly indicating a preventive or buffering effect of fruit and vegetable intake during early emotional development [23,24].

However, the lack of association with clinically significant depression could also reflect the low prevalence of this outcome in our sample, which may have limited the statistical power to detect associations of moderate magnitude.

Although no clear association was observed between fish consumption and total depression scores, higher daily intake of fish was linked to lower odds of clinical depression. This finding may reflect the well-established neurobiological effects of long-chain omega-3 fatty acids found in fish, such as eicosapentaenoic acid (EPA) and docosahexaenoic acid (DHA), which are known to influence brain structure, neurotransmitter activity, synaptic plasticity, and inflammatory processes [25,26]. This finding suggests that the influence of fish consumption on depressive symptoms may be subtle and only reaches statistical significance when the outcome is defined categorically (clinical depression), rather than when modeled as a continuous total score. Nevertheless, after correcting for multiple testing using the Benjamini–Hochberg false discovery rate adjustment, only vegetable consumption remained significantly associated with depression total scores, while no associations were confirmed for clinical depression or for other food groups, including fish. This pattern is most likely attributable to the very low prevalence of clinical outcomes in our sample, which limited the statistical power to detect associations of modest magnitude.

While evidence on children has only recently started to emerge, general population studies suggest that diets rich in anti-inflammatory and neuroprotective nutrients, found in fish, fruit, vegetables, and nuts, are associated with lower depression risk [27]. A systematic review also found that increased fruit and vegetable consumption is linked to reduced risk of depression among youth and young adults aged 15–45 years [28], supporting the potential value of early-life interventions.

Overall, the lack of associations between most other food groups and emotional outcomes suggests that, during early childhood, the relationship between diet and internalizing mental health outcomes may be more nuanced, potentially moderated by other behavioral and environmental factors.

### Strengths and Limitations

The main strengths of the present study are the use of a large birth cohort, the standardized assessments of emotional outcomes using the CBCL, and detailed dietary data derived from FFQs. The dual analysis of clinical classifications and continuous symptom scores allowed us to capture both overt and subthreshold manifestations of emotional distress, which is particularly relevant for identifying early risk factors and intervention targets.

However, several limitations warrant consideration. First and foremost, the cross-sectional design of the dietary and mental health assessments prevents any conclusions about the directionality or causality of the observed associations. Specifically, it is not possible to determine whether dietary patterns contributed to the emergence of emotional symptoms, or whether pre-existing emotional states influenced dietary behaviors (e.g., emotional eating, appetite changes). Nonetheless, recent evidence suggests that even short-term dietary exposures, as brief as several weeks, may influence emotional and behavioral outcomes in children, particularly during sensitive developmental periods [29,30]. Moreover, unmeasured confounding and bidirectional effects are likely in the context of complex behavioral and psychosocial processes, further limiting causal inference. Secondly, the food frequency questionnaire relied on parental recall and did not capture dietary diversity or food processing level (e.g., ultra-processed foods), which are important dimensions of diet quality. Moreover, we were not able to validate the FFQ using a 24 h dietary recall or food diary. However, FFQs are generally recognized for their acceptable validity and good reliability and are considered a suitable proxy for assessing dietary intake in preschool-aged children [31]. Thirdly, the study did not include biomarkers of nutrient status or inflammation, which could have strengthened biological plausibility. Fourthly, although our data was adjusted for sex and per capita income, several other factors such as parental mental health, screen time, and broader socioeconomic conditions can significantly influence both diet quality and psychological development [9,13,15]; the complexity of these interrelated influences may have reduced our ability to detect diet-specific effects, especially in a relatively healthy population with a narrow range of dietary habits. Fifthly, the sample’s generally low symptom burden may have limited our ability to detect associations of smaller magnitude. Finally, while response rates for the dietary questionnaire and the CBCL were comparable to those reported in other large-scale pediatric cohorts, they still may limit the generalizability of our findings, as selection bias cannot be entirely excluded, and the results should therefore be interpreted with appropriate caution.

## 5. Conclusions

Our findings support the hypothesis that higher intake of specific food groups, particularly vegetables, fruits and fish, is associated with better emotional health in preschool-aged children. While modest in effect size, these findings underscore the importance of promoting healthy dietary patterns from early childhood as part of a broader strategy for mental health prevention.

Further longitudinal research is needed to confirm these associations over time and to explore the underlying biological mechanisms. Interventions targeting early dietary habits may represent a feasible and modifiable pathway to improve long-term mental health outcomes in children.

## Figures and Tables

**Table 1:** Socio-demographic characteristics of the sample.

Variables	*n* = 1727
Age, median (IQR)	4.4 (4.2–4.5)
Female sex, *n* (%)	868 (50.3)
Income per capita, median (IQR)	687.4 (562.4–916.5)
Anxiety raw score, median (IQR)	3 (1–4)
Clinical anxiety, *n* (%)	52 (3.0)
Depression raw score, median (IQR)	1 (0–3)
Clinical depression, *n* (%)	38 (2.2)

**Table 2:** Robust linear regression coefficients with 95% CI for anxiety raw scores (**a**) and Odds Ratios (OR) with 95% Confidence Intervals (CI) for anxiety (**b**) ^a^.

	Raw Total Scores for Anxiety (a)			Clinical Anxiety (b)		
Variables (g/day)	Coeff.	95% CI	*p*-Value *	OR	95% CI	*p*-Value *
Fruit	−0.001	−0.003; 0.001	0.340	0.997	[0.991–1.003]	0.326
Vegetables	−0.003	−0.001; 0.0001	0.073	1.003	[0.996–1.010]	0.310
Legumes	0.008	−0.007; 0.022	0.489	1.016	[0.984–1.047]	0.296
Grains	0.002	−0.0001; 0.003	0.063	1.004	[1.001–1.007]	0.039
Milk	−0.0003	−0.001; 0.0003	0.424	1.000	[0.998–1.001]	0.957
Red meat	0.007	−0.002; 0.016	0.127	1.003	[0.981–1.025]	0.810
Cured meat	0.010	−0.011; 0.030	0.351	1.015	[0.965–1.069]	0.561
Fish	0.010	−0.0001; 0.020	0.052	1.008	[0.984–1.034]	0.507
Sweetened drinks	0.000	−0.0003; 0.001	0.492	1.000	[0.998–1.001]	0.839

^a^ All estimates are adjusted by sex and per capita income (EUR). * Significant *p*-values adjusted using the Benjamini–Hochberg procedure are marked with an asterisk (*).

**Table 3:** Robust linear regression coefficients with 95% CI for depression raw scores (**a**) and Odds Ratios (OR) with 95% Confidence Intervals (CI) for depression (**b**) ^a^.

	Raw Scores for Depression (a)			Clinical Depression (b)		
Variables (g/day)	Coeff.	95% CI	*p*-Value	OR	95% CI	*p*-Value
Fruit	−0.002	−0.003; −0.0001	0.031	0.999	[0.992–1.006]	0.737
Vegetables	−0.003	−0.005; −0.001	0.004 *	0.997	[0.987–1.007]	0.544
Legumes	−0.004	−0.014; 0.005	0.454	0.998	[0.957–1.041]	0.947
Grains	0.001	−0.001; 0.002	0.723	1.003	[0.999–1.007]	0.105
Milk	0.0002	−0.0003; 0.0006	0.467	1.000	[0.999–1.001]	0.967
Red meat	0.004	−0.003; 0.011	0.275	1.018	[0.993–1.040]	0.131
Cured meat	0.009	−0.007; 0.025	0.272	1.046	[0.999–1.095]	0.051
Fish	−0.003	−0.010; 0.004	0.365	0.951	[0.907–0.997]	0.036
Sweetened drinks	0.003	−0.0001; 0.001	0.108	1.000	[0.999–1.001]	0.314

^a^ All estimates are adjusted by sex and per capita income (EUR). * Significant *p*-values adjusted using the Benjamini–Hochberg procedure are marked with an asterisk (*).

## Data Availability

Due to the sensitive nature of the questions asked in this study, Piccolipiù survey respondents were assured raw data would remain confidential and would not be shared. For this reason, the authors do not have permission to share data.

## Supplementary Materials

The following supporting information can be downloaded at https://www.mdpi.com/article/10.3390/nu17182909/s1: Figure S1. Flowchart of study participants. Table S1. Food group consumption (g/day) by clinical anxiety and clinical depression. Table S2. Univariate robust linear regression coefficients with 95% CI for anxiety and depression raw scores.

## Abbreviations

The following abbreviations are used in this manuscript:

GLAD	Global burden of disease Lifestyle And mental Disorders
CMD	Common Mental Disorders
GBD	Global Burden of Disease
CBCL	Child Behavior Checklist
FFQ	Food Frequency Questionnaire
IQR	InterQuartile Range
OR	Odds Ratio
CI	Confidence Interval
EPA	EicosaPentaenoic Acid
DHA	DocosaHexaenoic Acid
