# Supplementary material for: Association Between Diet and Emotional Symptoms in Early Childhood: Cross-Sectional Results from the Piccolipiù Cohort
Source: Nutrients. 2025 Sep 9;17(18):2909. doi: 10.3390/nu17182909 (PMC12472278; doi:10.3390/nu17182909)
Supplement: Supplementary file 1 [file nutrients-17-02909-s001.zip › nutrients-3821854-supplementary.pdf]

Figure S1. Flowchart of study participants.

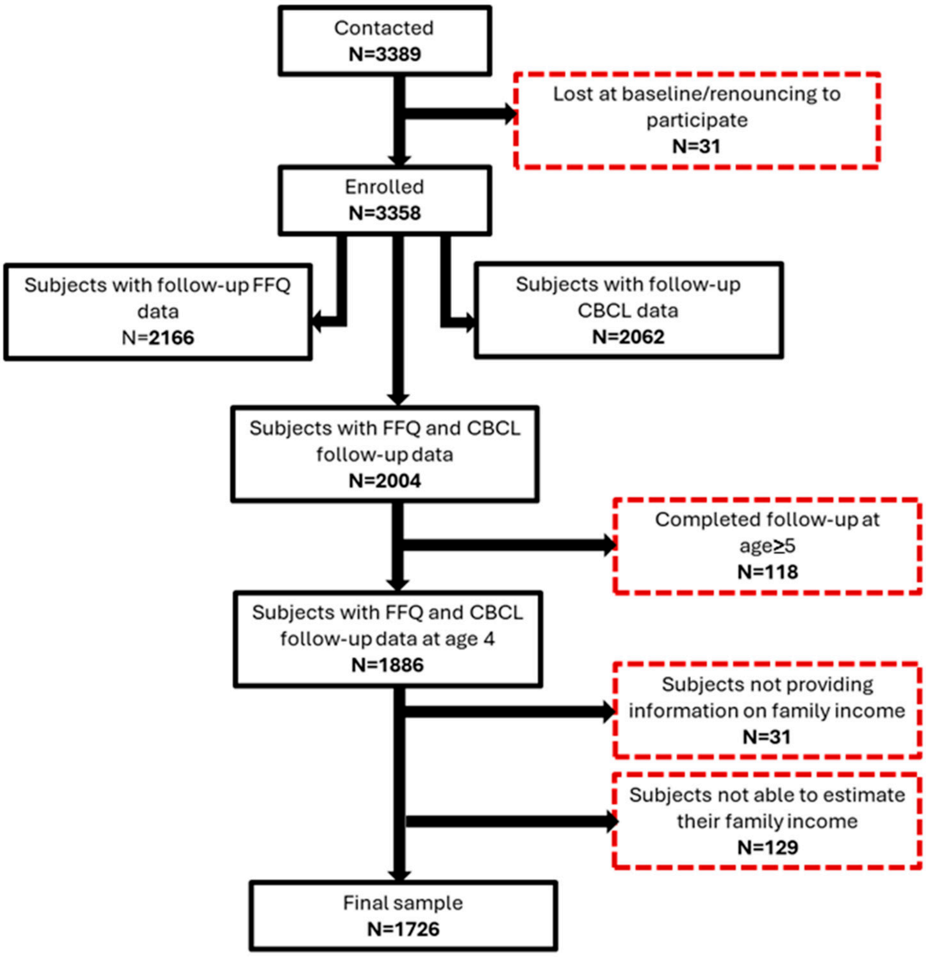

Table S1. Food group consumption (g/day) by clinical anxiety and clinical depression[a].

	Clinical anxiety				Clinical depression			
Variables (g/day)	No n=1674	Yes n=52	OR [95% CI]	p-value	No n=1688	Yes n=38	OR [95% CI]	p-value
Fruit	150 (100-180)	125 (100-180)	0.997 [0.994-1.004]	0.401	150 (100-180)	150 (100-180)	0.999 [0.992-1.006]	0.817
Vegetables	50 (29-86)	50 (29-100)	1.003 [0.996-1.010]	0.340	50 (29-86)	43 (21-71)	1.000 [0.987-1.007]	0.529
Legumes	10 (6-17)	8 (6-17)	1.016 [0.986-1.048]	0.296	10 (6-15)	10 (6-17)	0.999 [0.959-1.042]	0.995
Grains	162 (125-208)	190 (133-247)	1.004 [1.001-1.007]	0.028	161 (124-208)	186 (136-233)	1.003 [0.999; 1.007]	0.111
Milk	200 (71-286)	200 (33-300)	1.000 [0.999 -1.001]	0.987	200 (71-286)	200 (43-300)	1.000 [0.999-1.001]	0.916
Red meat	17 (10-26)	20 (11-26)	1.001 [0.978-1.023]	0.950	17 (10-26)	21 (11-31)	1.016 [0.998-1.002]	0.156
Cured meat	6 (3-9)	9 (4-11)	1.009 [0.954-1.054]	0.722	6 (3-9)	9 (3-11)	1.042 [0.997-1.089]	0.067
Fish	14 (7-20)	14 (10-21)	1.005 [0.979-1.032]	0.687	14 (7-20)	12 (8-14)	0.946 [0.903-0.992]	0.023

Sweetened drinks	71 (0-200)	86 (29-200)	1.000 [0.999-1.001]	0.972	85 (0.2-200)	100 (0.4-200)	1.000 [0.999-1.001]	0.271

[a]Results are reported as median and interquartile range (IQR), and unadjusted odds ratios (OR) with 95% confidence intervals (CI).

*Significant p-values adjusted using the Benjamini-Hochberg procedure are marked with an asterisk (*).

Table S2. Univariate robust linear regression coefficients with 95% CI for anxiety and depression raw scores.

	Anxiety			Depression		
Variables (g/day)	Coeff.	95% CI	p-value	Coeff.	95% CI	p-value
Fruit	-0.0003	-0.002; 0.002	0.738	-0.002	-0.003; 0.0001	0.036*
Vegetables	-0.004	-0.007; -0.001	0.009	-0.003	-0.006; -0.001	0.001
Legumes	0.005	-0.009; 0.019	0.431	-0.003	-0.013; 0.006	0.494
Grains	0.001	-0.0001; 0.003	0.068	-0.0004	-0.001; 0.001	0.466
Milk	-0.0002	-0.001; 0.0004	0.445	0.0001	-0.0002; 0.0008	0.525
Red meat	0.006	-0.003; 0.015	0.094	0.002	-0.004; 0.008	0.497
Cured meat	0.008	-0.011; 0.028	0.376	0.003	-0.011; 0.017	0.711
Fish	0.009	-0.001; 0.020	0.082	-0.001	-0.008; 0.006	0.735
Sweetened drinks	0.0002	-0.0002; 0.001	0.567	0.0003	-0.00005; 0.0008	0.159

*Significant p-values adjusted using the Benjamini-Hochberg procedure are marked with an asterisk (*).
